# Supplementary material for: The Dual-Task Cost Is Due to Neural Interferences Disrupting the Optimal Spatio-Temporal Dynamics of the Competing Tasks
Source: Front Behav Neurosci. 2021 Aug 19;15:640178. doi: 10.3389/fnbeh.2021.640178 (PMC8416616; doi:10.3389/fnbeh.2021.640178)
Supplement: Supplementary file 1 [file Data_Sheet_1.PDF]

TABLES

Table I

Patient ID	Age	Sex	Handedness	Seizure Onset Zone	Drug treatment	Education
P1	15	F	R	Right insulo-temporal	NA	NA
P2	25	M	R	Right fronto-temporal	NA	Bachelor degree
P3	.32	M	Unknown	Bilateral temporal	NA	High school graduate
P4	37	M	R	Left insular – orbitofrontal	NA	High school graduate
P5	21	M	R	Left fronto-temporal	NA	Undergraduate studies
P6	34	F	R	Left insulo-temporal	NA	High school graduate
P7	26	F	R	Right frontal cryptogenic	NA	Bachelor degree
P8	15	F	L	Left temporal infero-basal	NA	NA
P9	48	F	Amb	Bilateral fronto-temporal	NA	Unknown
P10	58	M	R	Left fronto-temporal	NA	Uncompleted high school
P11	21	F	L	Left temporo-mesial	NA	Bachelor degree
P12	49	M	L	Right temporal antero-inferior	NA	Uncompleted high school

Table I. Clinical/Sociodemographic data. We did not include the medical treatment at the time because each patient has a mobile treatment, and if it is not possible to know the precise scheme was during the day of the intervention, we preferred to omit it.

Table II

Patient ID	iEEG site	X	Y	Z	Freesurfer labelization
P1	R9	67	0	10	Subcentral gyrus
P1	F6	41	-50	-13	Lateral Occipito-Temporal Sulcus
P2	M9	38	8	33	Inferior Frontal Sulcus
P3	X7	34	25	2	Anterior Insula
P3	X’9	-30	23	5	Anterior Insula
P4	R’4	-44	7	16	Inferior part of the Precentral Sulcus
P4	R’9	-61	7	16	Precentral Cortex
P4	G’8	-33	20	20	Inferior Frontal Sulcus
P4	G’13	-45	33	21	Inferior Frontal Sulcus
P4	F’9	-28	44	30	Middle Frontal Sulcus
P4	Y’13	-31	50	27	Middle Frontal Gyrus
P4	E’7	-50	-37	-21	Basal Temporal Cortex
P5	S’9	-41	0	40	Precentral Sulcus

P5	S’12	-54	0	41	Precentral Gyrus
P5	K’10	-42	35	12	Inferior Frontal Sulcus
P5	Z’2	-11	19	46	Anterior Cingulate Gyrus/Sulcus
P5	L’3	-36	-52	-11	Lateral Occipito-Temporal Sulcus
P6	D’7	-55	-42	8	Superior Temporal Sulcus
P7	E4	41	17	4	Anterior Insula
P7	S2	11	16	52	Anterior Cingulate Gyrus/Sulcus
P9	G’12	-45	41	14	Middle Frontal Gyrus
P11	Q’2	-40	25	14	Inferior Frontal Sulcus
P11	X5	30	20	-6	Anterior Insula
P11	X’9	-30	23	5	Anterior Insula
P11	C’12	-60	-37	-12	Middle Temporal Gyrus
P12	X7	33	17	10	Anterior Insula
P12	A9	52	-3	-20	Middle Temporal Gyrus

Table II. MNI-coordinates for each site displayed in the figures with the anatomical labelization provided by the Freesurfer software (corrected when obviously inaccurate) (https://surfer.nmr.mgh.harvard.edu).
