# Supplementary material for: The Dual-Task Cost Is Due to Neural Interferences Disrupting the Optimal Spatio-Temporal Dynamics of the Competing Tasks
Source: Front Behav Neurosci. 2021 Aug 19;15:640178. doi: 10.3389/fnbeh.2021.640178 (PMC8416616; doi:10.3389/fnbeh.2021.640178)
Supplement: Supplementary file 2 [file Data_Sheet_2.PDF]

The verbal localizer task and verbal working memory tasks that we used as localizers are designed to identify neural population supporting phonological/semantic processes and verbal working memory maintenance, respectively. Therefore, we have full confidence - by design - that iEEG sites active specifically in those conditions - and inactive or with much weaker activation during a visual discrimination condition, for instance - support a verbal type of processing (see the example of S’9 down below). According to our proposed scenario, S'12 in the precentral gyrus of P5 should also have a reduced response in the DT condition, but if it was only supporting verbal processing as well. Yet, a detailed comparison of the response of S'9 and S'12 (see the example of S’12 down below) shows a strikingly dissimilar response patterns in S’9 and S’12 : as can be seen, the response in S'9 is largely specific of language processes while the response in S'12 is general and occurs as soon as a visual stimulus must be processed. As any new strategy found by P5 to perform BLAST would still require active processing of visual stimuli, it should trigger a response in S'12, as shown here. In contrast, the response in S'9 would only be visible if the participant relies on a verbal strategy.

Figure A

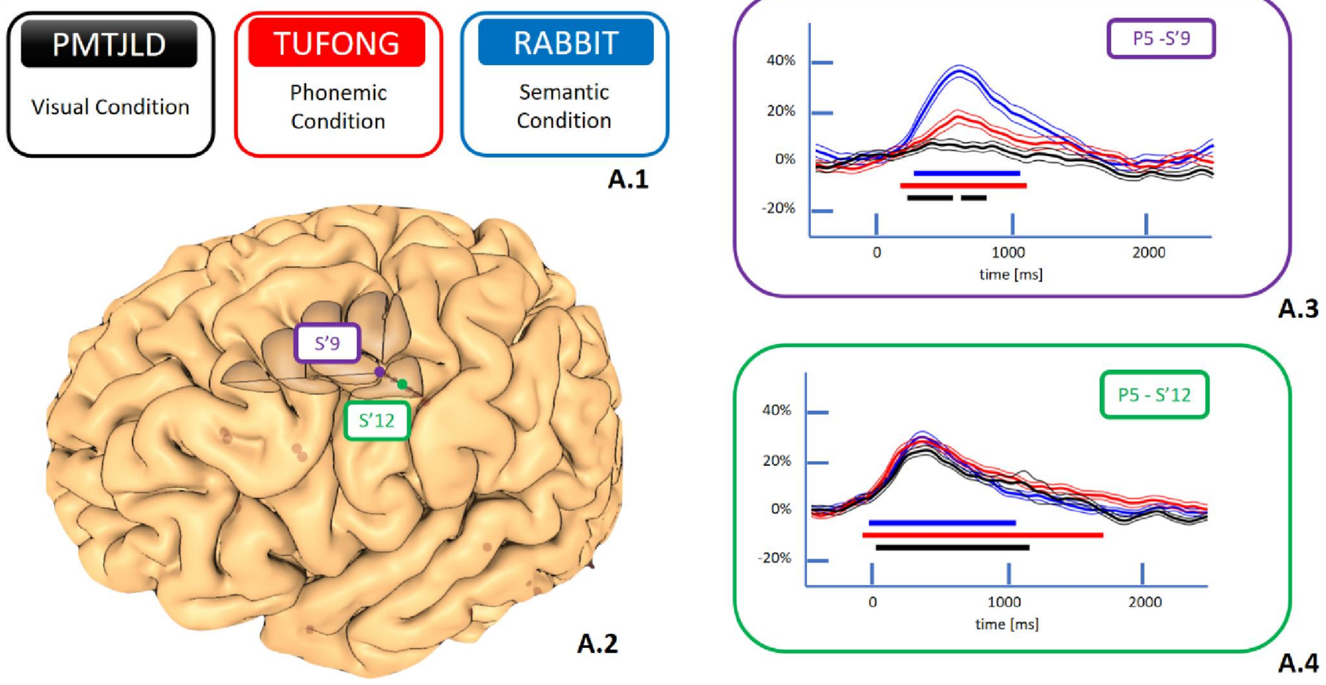

Figure A. *On this experiment, the patient (P5) must recognize if a six letters simultaneous character string (A.1) forms:*

1) *a random consonant string (“Visual condition”)*
2) *pseudo words with phonemic content (“Phonemic condition”)*
3) *or a word with semantic content such as a living entity (“Semantic condition”), via a button press*

The purple and green dots (A.2) show sites S’9 and S’12, respectively, with a specific response to Semantic stimuli in S’9 (A.3) and no evidence of language specific process in S’12 (A.4). In the last two figures, the X-axis corresponds to time (with the stimulus shown at 0 ms), and the y-axis corresponds to HFA energy increase expressed in % of the mean HFA level across the entire experiment. Horizontal bars indicate a significant energy increase in the [50-150 Hz] range relative to a prestimulus baseline.

If the insula was indeed specifically involved in verbal functions, then it should decrease its activity in the DT condition of T1 if the patient stopped using a verbal strategy, and this would contradict our interpretation of the middle temporal gyrus data. However, the anterior insula is not related to language loops, as evident in Figure B which shows a strong activation for two illustrative sites (X’9 [P3] and E4 [P7] of figure 6) during a visuo-spatial working memory task with no verbal component. This fits well with the current interpretation that the anterior insula is involved in domain general processes (in particular, cognitive control) (Shashidhara et al, 2019). In addition, figure B.3 shows the responses in the dorsal anterior insula (dAI) in a separate patient who performed a bedside Verbal Fluency Test, part of a, pencil and paper, neuropsychological investigation. Although the paradigm was not computerized, a clear responsivity of the dAI to the VFT task is visible, which is very systematic from this region when that exam is performed. This leads us to believe that the dAI is a common node of the BLAST and the VFT networks.

Figure B

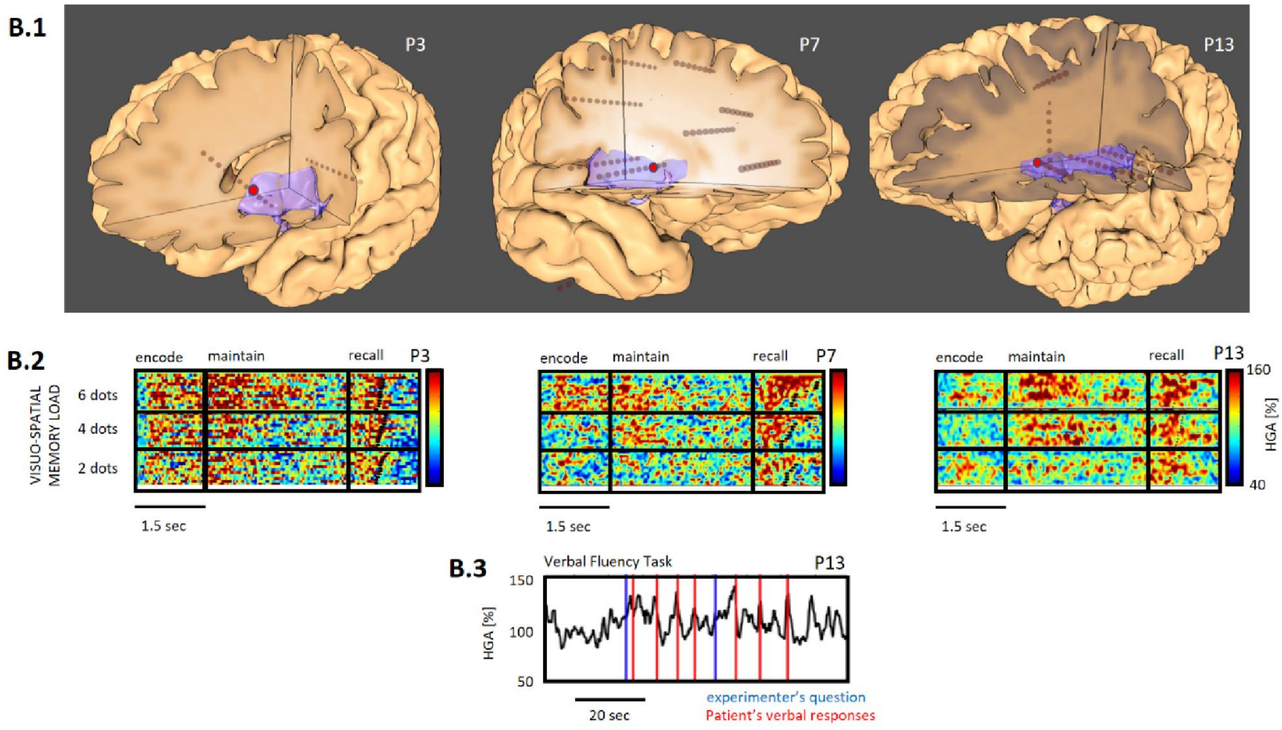

Figure B. *Non-verbal responses in the dorsal anterior insula (dAI), and responses to a VFT in a single-task condition.*

Despite P13 did not perform BLAST in ST/DT condition, P3, P7 and P13 performed a non-verbal visuo- spatial working memory task where they observed a 4x4 checkerboard. On it, 2, 4 or 6 points are presented at random, setting in total three different conditions (easy, moderate or hard, respectively). The dotted checkboard is shown first during 1500 ms (encode), then the dots disappear during 3000 ms (maintain), and finally a single dot appears, and patients must confirm whether or not the dot was part of the original ones (recall) via a button press. Each brain plots the position of an electrode (red dot) at our site of interest; in this case the dAI of each patient (B.1). Matrixes display the variation of HGA (B.2), in % induced by the task for all trials (y axis) as a function of time (x axis) (100% = average HGA during the entire experiment). Responses in this task clearly indicate a participation in non-verbal functions. Sites for P3 (X’9) and P7 (E4) are also shown in figure 6.

Additionally, P13 performed a bedside Verbal Fluency Test in single-task condition. The VFT graph (B.3) illustrates a clear coincidence between verbal responses and activity peaks in the dAI.
